# Dietary Lysine Levels Improved Antioxidant Capacity and Immunity *via* the TOR and p38 MAPK Signaling Pathways in Grass Carp, *Ctenopharyngodon idellus* Fry

**DOI:** 10.3389/fimmu.2021.635015

**Published:** 2021-02-25

**Authors:** Dongyu Huang, Sahya Maulu, Mingchun Ren, Hualiang Liang, Xianping Ge, Ke Ji, Heng Yu

**Affiliations:** ^1^ Wuxi Fisheries College, Nanjing Agricultural University, Wuxi, China; ^2^ Key Laboratory for Genetic Breeding of Aquatic Animals and Aquaculture Biology, Freshwater Fisheries Research Center (FFRC), Chinese Academy of Fishery Sciences (CAFS), Wuxi, China

**Keywords:** grass carp (*Ctenopharyngodon idellus*), lysine, antioxidant capacity, immunity, TOR signaling pathway, p38 MAPK signaling pathway

## Abstract

An 8-week rearing trial was designed to appraise the dietary lysine levels on intestinal antioxidant capacity and immunity of grass carp fry. Six practical diets were prepared with graded levels of lysine (1.44, 1.79, 1.97, 2.44, 2.56 and 2.87% dry matter), and these diets were fed to grass carp fry. The results showed that the activities of intestinal antioxidant factors including catalase and glutathione peroxidase were markedly improved by the 2.44% dietary lysine compared with the control diet (1.44% dietary lysine) (*P* < 0.05). In terms of antioxidants, compared with the control diet, the 2.44% diet markedly upregulated the mRNA expression levels of target of rapamycin, S6 kinase1 and nuclear factor erythroid 2-related factor 2 pathway-related antioxidant genes, containing catalase and glutathione peroxidase 1*α* (*P* < 0.05) and downregulated the mRNA levels of Kelch-like ECH-associated protein 1 (*P* > 0.05). The mRNA levels of 4E-binding protein 2 showed the opposite trend compared with those of target of rapamycin, and the minimum value was observed in the group of 1.97% dietary lysine (*P* < 0.05). In terms of immunity, compared with the 1.44% diet, the 2.44% diet markedly suppressed the intestinal p38 mitogen-activated protein kinase and interferon *γ*2 mRNA levels (*P* < 0.05). Moreover, nuclear factor-kappa B p65, tumor necrosis factor α, interleukin 6, interleukin 8, and interleukin 15 mRNA levels all exhibited the same trend as p38 mitogen-activated protein kinase and interferon *γ*2; however, the difference among all the lysine treatments groups was not significant (*P* > 0.05). The anti-inflammatory cytokines transforming growth factor *β*2 and interleukin 4/13B mRNA levels in the intestine were remarkably upregulated by high dietary lysine levels (2.56 and 2.87%) (*P* < 0.05), and when the dietary lysine level reached 2.44%, the interleukin 4/13A mRNA levels were strikingly increased (*P* < 0.05). Overall, the data suggested that 2.44% dietary lysine could strengthen the immune and antioxidant capacities of grass carp fry *via* activating the target of rapamycin (TOR) signaling pathway, and suppressing the p38 mitogen-activated protein kinase (p38 MAPK) signaling pathway, which then improve the survival rate.

## Introduction

In animals, as an essential amino acid, lysine is important for body growth performance (1). Previous studies also reported that dietary lysine levels can regulate metabolism and immunity in mammals (2). Similar to its role in mammals, lysine also acts a pivotal part in fish and can enhance the growth and feed efficiency of fish, such as Indian major carp (*Cirrhinus mrigala*) (3), tilapia (*Oreochromis niloticus*) (4), and large yellow croaker (*Pseudosciaena crocea* R) (5). In addition, lysine is closely related to the activity of the fish immune system, and appropriate lysine levels can modify immune response (6).

In fish, non-specific immunity is the first line of defense against the invasion of foreign matter. Non-specific immunity mainly includes active immune substances and cytokines secreted by the intestine (7). As one of the mitogen-activated protein kinase (MAPK) family, p38 mitogen-activated protein kinase (p38 MAPK) participates in the regulation of cellular processes, including inflammation and apoptosis (8). Qin et al. (9) reported that activated MAPKs can trigger the stimulation of other kinase targets, which then activate the transcription of proinflammatory genes. Moreover, nuclear factor kappa B (NF-*κ*B), as the downstream target of the p38 MAPK signaling pathway, is a major signaling molecule that regulates the production of cytokines (10, 11). Several studies have shown that appropriate dietary lysine levels can improve immunity in aquatic animals, and that deficiency of dietary lysine may activate the NF-*κ*B, thereby upregulating the expression of proinflammatory cytokines and downregulating the expression of anti-inflammatory cytokines (12–14). Previous studies mostly focused on the effect of the NF-*κ*B signaling pathway on inflammatory factors, but ignored the regulatory effect of the upstream molecule p38 MAPK on NF-*κ*B. Therefore, it is important to explore the effect of lysine on immunity through the p38 MAPK/NF-*κ*B signaling pathway.

On the other hand, fish possess effective antioxidant systems that prevent oxidative damage (15), for example the antioxidant enzymes catalase (CAT), glutathione peroxidase (GPx), and superoxide dismutase (SOD), as well as the low-molecular-weight antioxidant glutathione (GSH) (16). The activities of the antioxidant enzymes mentioned above, similar to the expression of the corresponding antioxidant genes, can be regulated by nuclear factor erythroid 2-related factor 2 (Nrf2) (17). Li (18) reported that lysine can increase the activities of antioxidant enzymes by upregulating Nrf2, and upregulating the expression of antioxidant enzyme genes, thereby increasing the ability of free radical scavenging to protect against oxidative damage. Additionally, Memon et al. (19) mentioned that MAPKs can indirectly regulate the Nrf2 signaling pathway to neutralize oxidative stress, but the mechanism is still poorly understood. In fish species, the mechanism by which dietary lysine affects antioxidant capacity by modulating the p38 MAPK/Nrf2 signaling pathway, which is worthy of investigation.

P38 MAPK is related to the regulation of immune and antioxidant capacities, and the target of rapamycin (TOR) signaling molecule is involved in this process. Thomson et al. (20) pointed out that the TOR signaling pathway is closely related to inflammatory stimuli. TOR regulates two important translation regulators namely, ribosomal protein S6 kinase 1 (S6K1) and eukaryotic translation initiation factor 4E-binding proteins (4E-BPs) (21), and the inhibition of TOR can upregulate the mRNA expression of proinflammatory cytokines (22). In addition, TOR is the upstream signaling molecule of Nrf2 (23). Thus, the TOR signaling pathway has also been considered to be an integration point of antioxidant capacity and the immune system, and it is meaningful to evaluate the effect of lysine on the TOR signaling pathway.

As one of the main farmed aquaculture species in China, grass carp (*Ctenopharyngodon idella*) has great commercial value and is widely distributed worldwide (24). To date, previous studies have confirmed that optimum dietary lysine levels can improve the growth performance of grass carp at different stages of growth (18, 25–27). However, in terms of antioxidant activity and immunity, previous studies only reported on the adult stage (28). Furthermore, fish in the fry stage (less than 1 g) have a much higher mortality rate than those in the adult stage (29), and the resistance of fry to external pathogenic microorganisms mainly depends on their own non-specific immunity (30); therefore, it is more important to study the immunity and antioxidation mechanisms in the fry stage for improving the survival rate. In addition, many scholars have begun to evaluate the immune and antioxidant capabilities of grass carp by the TOR (31–34) or p38 MAPK (35–37) signaling pathway in recent years, but knowledge of the effects of dietary lysine levels on the antioxidant capacity and immunity of grass carp fry is still limited. Hence, this study was aimed to investigate the effects of dietary lysine levels on the immune capacity and antioxidant status, regulated *via* the TOR and p38 MAPK signaling pathways in grass carp fry.

## Materials and Methods

### Diets

Diets with six different lysine levels (1.44% (control group), 1.79, 1.97, 2.44, 2.56, and 2.87% dry matter) were designed. In the experimental diets, the main protein sources and lipid source are listed in **Table 1**, and the amino acid premix of each diet was listed in our previous report (27). The steps of feed pelleting (including crushing, sieving, weighing, mixing, granulating, *etc*.) were consistent with that described by Ren et al. (34), and then all the diets were dried in the oven (45°C). Finally, the diets were kept in a −20°C freezer for future use.

**Table 1:** Composition of the basal diet.

Ingredients (g/kg)	
Fish meal^a^	50
Rapeseed meal^b^	190
Soybean meal^c^	340
Wheat flour^d^	189.1
Soybean oil	30
Choline chloride	1
Vitamins premix^e^	10
Mineral premix^f^	10
Calcium dihydrogen phosphate	25
Amino acid premix^g^	64.8
Rice bran	50
Ethoxy quinoline	0.1
Glycine^h^	20-*
L-lysine^i^	*
Bentonite	20

The feed formulation references Huang et al. (27). *means increased additive lysine levels (0, 4.0, 8.0, 12.0, 16.0, and 20.0 g/kg, respectively) of six diets

a Fish meal, obtained from obtained from Wuxi Tongwei feedstuffs Co., Ltd, Wuxi, China, crude protein 65.8%, crude lipid 9.5%.

b Rapeseed meal, obtained from Wuxi Tongwei feedstuffs Co., Ltd, Wuxi, China, crude protein 39.2%, crude lipid 6.1%.

c Soybean meal, obtained from Wuxi Tongwei feedstuffs Co., Ltd, Wuxi, China, crude protein 50.8%, crude lipid 4.3%.

d Wheat flour, obtained from Wuxi Tongwei feedstuffs Co., Ltd, Wuxi, China, crude protein 13.1%, crude lipid 4.0%.

e Vitamins premix (IU, or mg/kg of diet): vitamin A, 25,000 IU; vitamin D3, 20,000 IU; vitamin E, 200 mg; vitamin K3, 20 mg; thiamin, 40 mg; riboflavin, 50 mg; calcium pantothenate, 100 mg; pyridoxine HCl, 40 mg; cyanocobalamin, 0.2 mg; biotin, 6 mg; folic acid, 20 mg; niacin, 200 mg; inositol, 1,000 mg; vitamin C, 2,000 mg; choline, 2,000 mg.

f Mineral premix (g/kg of diet): calcium biphosphate, 20 g; sodium chloride, 2.6 g; potassium chloride, 5 g; magnesium sulphate, 2 g; ferrous sulphate, 0.9 g; zinc sulphate, 0.06 g; cupric sulphate, 0.02 g; manganese sulphate, 0.03 g; sodium selenate, 0.02 g; cobalt chloride, 0.05 g; potassium iodide, 0.004 g.

g Amino acid premix (L-form, g/kg dry diet): histidine, 3.1; isoleucine, 3.3; leucine, 7.3; methionine, 4.5; phenylalanine, 2.0; threonine, 2.5; valine, 4.1; tryptophan, 0.2; aspartic acid, 7.0; glycine, 13.8; alanine, 9.6; proline, 7.5. Amino acids obtained from Feeer Co., LTD (Shanghai, China).

h Glycine, obtained from Feeer Co., LTD (Shanghai, China).

i L-lysine, obtained from Feeer Co., LTD (Shanghai, China).

**Table 2:** The chemical analysis used in the experiment.

Items	Methods	Assay kits/Testing equipment
*Composition of ingredients*		
Amino acids (except tryptophan)	Acid hydrolysis	Amino acid analyzer: SYKAM S-433D (Sykam GmbH, Munich, Germany)
Tryptophan	Alkali hydrolysis	
*Intestinal parameters related antioxidant capacity*		
SOD^a^	WST-1 method	Assay kits purchased from Jian Cheng Bioengineering Institute (Nanjing, China); Spectrophotometer (Thermo Fisher Multiskan GO, Shanghai, China).
GSH^b^	Microplate method	
GPx^c^	Colorimetric method	
MDA^d^	TBA method	
CAT^e^	Ammonium molybdenum acid method	

a SOD, Superoxide dismutase.

b GSH, Glutathione.

c GPx, Glutathione peroxidase.

d MDA, Malondialdehyde.

e CAT, Catalase.

### Experimental Procedure

Experimental healthy (lively; no scars on the whole body; no bleeding symptoms) and similarly sized grass carp fry (initial weight 0.36 ± 0.00 g) were from the breeding farm of the Freshwater Fisheries Research Center of the Chinese Academy of Fishery Sciences, and placed in tanks (180 L), in order to adjust to the breeding condition (photoperiod: the same as natural light, pH: 7.0−7.8, water temperature: 27 ± 2°C, and dissolved oxygen: 6.8 ± 0.8 mg/L). After two weeks, 720 fish were chosen and randomly divided into 18 tanks (triplicate tanks) with 40 fish in each tank in a water circulation system culture for 8 weeks. Throughout the entire experiment, the fish were hand-fed four times daily at a feeding rate of 5% their weight. The fish were counted and weighed every 2 weeks from the onset of the experiment trial to check the body weight and to adjust the amount of feed. At the same time, the number of fish death was recorded per tank. The handling of the fry was approved by the Institutional Animal Care and Ethics Committee of Nanjing Agricultural University, Nanjing, China. [Permit number: SYXK (Su) 2011-0036].

### Sample Collection and Analysis

After the fish were fasted for 20 h, three intestinal samples were sampled from each tank at the end of the rearing trial. The intestinal samples were frozen in −80°C refrigerator for later analysis.

The amino acid concentration of ingredients and the intestinal activities of antioxidant factors were analyzed according to our previous study (38). The main testing equipment, methods, and kits are shown in **Table 2**.

**Table 3:** Primer sequence for qRT-PCR.

Target genes	Forward primer (5’-3’)	Reverse primer (5′–3′)	Temperature (°C)	Accession no.
*β*-actin	CGTGACATCAAGGAGAAG	GAGTTGAAGGTGGTCTCAT	61.4	M25013
p38 MAPK^a^	TGGGAGCAGACCTCAACAAT	TACCATCGGGTGGCAACATA	60.4	KM112098
TOR^b^	TCCCACTTTCCACCAACT	ACACCTCCACCTTCTCCA	61.4	JX854449
S6K1^c^	TGGAGGAGGTAATGGACG	ACATAAAGCAGCCTGACG	54.0	EF373673
4EBP1^d^	GCTGGCTGAGTTTGTGGTTG	CGAGTCGTGCTAAAAAGGGTC	60.3	KT757305
4EBP2^e^	CACTTTATTCTCCACCACCCC	TTCATTGAGGATGTTCTTGCC	60.3	KT757306
NF-kB p65^f^	GAAGAAGGATGTGGGAGATG	TGTTGTCGTAGATGGGCTGAG	62.3	KJ526214
Nrf2^g^	CTGGACGAGGAGACTGGA	ATCTGTGGTAGGTGGAAC	62.5	KF733814
Keap1^h^	TTCCACGCCCTCCTCAA	TGTACCCTCCCGCTATG	63.0	KF811013
IL-4/13A^i^	CTACTGCTCGCTTTCGCTGT	CCCAGTTTTCAGTTCTCTCAGG	55.9	KT445871
IL-4/13B^j^	TGTGAACCAGACCCTACATAACC	TTCAGGACCTTTGCTGCTTG	55.9	KT625600
TGF-*β*2^k^	TACATTGACAGCAAGGTGGTG	TCTTGTTGGGGATGATGTAGTT	55.9	KM279716
IFN*-γ*2^l^	TGTTTGATGACTTTGGGATG	TCAGGACCCGCAGGAAGAC	60.4	JX657682
TNF-α^m^	CGCTGCTGTCTGCTTCAC	CCTGGTCCTGGTTCACTC	58.4	HQ696609
IL-6^n^	CAGCAGAATGGGGGAGTTATC	CTCGCAGAGTCTTGACATCCTT	62.3	KC535507.1
IL-8^o^	ATGAGTCTTAGAGGTCTGGGTG	ACAGTGAGGGCTAGGAGGG	60.3	JN663841
IL-15^p^	CCTTCCAACAATCTCGCTTC	AACACATCTTCCAGTTCTCCTT	61.4	KT445872
CAT^q^	GAAGTTCTACACCGATGAGG	CCAGAAATCCCAAACCAT	58.7	FJ560431
Cu/Zn-SOD^r^	CGCACTTCAACCCTTACA	ACTTTCCTCATTGCCTCC	61.5	GU901214
GPx-1*α* ^s^	GGGCTGGTTATTCTGGGC	AGGCGATGTCATTCCTGTTC	61.5	EU828796
GPx-4^t^	CTGGAGAAATACAGGGGTTACG	CTCCTGCTTTCCGAACTGGT	60.3	KU255599

a p38 MAPK, p38 mitogen-activated protein kinase.

b TOR, Target of rapamycin.

c S6K1, S6kinase1.

d 4EBP1, 4E binding protein 1.

e 4EBP2, 4E binding protein 2.

f NF-kB p65, Nuclear factor-kappa B p65.

g Nrf2, Nuclear factor erythroid 2-related factor 2.

h Keap1, Kelch-like ECH-associated protein 1.

i IL-4/13A, Interleukin 4/13A.

j IL-4/13B, Interleukin 4/13B.

k TGF-β2, Transforming growth factor β2.

l IFN-γ2, Interferon γ2.

m TNF-α, Tumour necrosis factor α.

n IL-6, Interleukin 6.

o IL-8, Interleukin 8.

p IL-15, Interleukin 15.

q CAT, Catalase.

r Cu/Zn-SOD, Copper zinc superoxide dismutase.

s GPx-1α, Glutathione peroxidase 1α.

t GPx-4, Glutathione peroxidase 4.

The relative mRNA expression levels of target genes were tested by quantitative real-time PCR (qRT-PCR) analysis. First, the RNA was extracted from the intestines by RNAiso plus kits, then evaluating the quality and quantity of RNA by Nano Drop 2000 spectrophotometer (Thermo Fisher Multiskan GO, Shanghai, China), and finally operating the reaction system using the One Step SYBR^®^ PrimeScript^®^ Plus RT-PCR Kits (Cat# RR096A) and the specific primers of target genes (**Table 3**) on a 7500 real-time PCR machine (Applied Biosystems, Carlsbad, USA). *β*-actin was used as the internal reference gene in this study, and no significant changes were observed (39). At last, the mRNA expression levels of the antioxidant and immune genes were determined through the Pfaffl’s mathematical model (40).

**Table 4:** Effects of dietary lysine levels on intestinal antioxidant parameters of grass carp fry (means ± S.E.M.)^a^ .

Lysinelevels (%)	CAT^b^ (U/mg protein)	SOD^c^ (U/mg protein)	MDA^d^ (nmol/mg protein)	GSH^e^ (μmol/g protein)	GPx^f^ (U/mg protein)
1.44	1.10 ± 0.30^a^	0.38 ± 0.04	1.13 ± 0.37	8.65 ± 1.49	16.19 ± 1.71^a^
1.79	1.70 ± 0.54^a^	0.38 ± 0.02	1.09 ± 0.32	7.73 ± 1.52	24.24 ± 2.43^a^ ^b^
1.97	3.06 ± 0.43^a^ ^b^	0.49 ± 0.02	1.00 ± 0.44	12.10 ± 1.82	24.59 ± 4.66^a^ ^b^
2.44	3.92 ± 0.70^b^	0.48 ± 0.06	0.99 ± 0.20	13.46 ± 1.74	35.52 ± 7.51^b^
2.56	2.57 ± 0.40^a^ ^b^	0.43 ± 0.03	0.98 ± 0.40	13.54 ± 2.17	36.51 ± 2.33^b^
2.87	1.32 ± 0.30^a^	0.37 ± 0.02	1.01 ± 0.13	9.90 ± 2.20	22.86 ± 4.59^a^ ^b^

a All data are means of triplicate, values in the same column with different superscripts are significantly different (P < 0.05).

b CAT, Catalase.

c SOD, Superoxide dismutase.

d MDA, Malondialdehyde.

e GSH, Glutathione.

f GPx, Glutathione peroxidase.

### Statistics Analysis

All data were analyzed by one-way ANOVA in the SPSS 24.0 statistical software package, and presented as the means ± S.E.M. Tukey’s multiple comparisons were used to examine the differences between the groups, differences were considered significant when *P* < 0.05.

## Results

### Intestinal Antioxidant Factors

The intestinal antioxidant factor results are shown in **Table 4**. The CAT activity increased with the dietary lysine levels increased from 1.44 to 2.44%, and then decreased (*P* < 0.05). The GPx activity in the fish fed the 2.44 and 2.56% lysine diets was remarkably higher than the control group (1.44% dietary lysine) (*P* < 0.05). The activities of both SOD and GSH showed the same trend of first increasing and then decreasing; however, there were no obvious changes among all the treatment groups (*P* > 0.05). Moreover, the MDA contents showed an opposite trend compared with the antioxidant enzymes, but no obvious changes were observed in the fish fed different dietary lysine levels (*P* > 0.05).

### Relative mRNA Expression of Genes Related to the Target of Rapamycin Signaling Pathway

**Figure 1:**
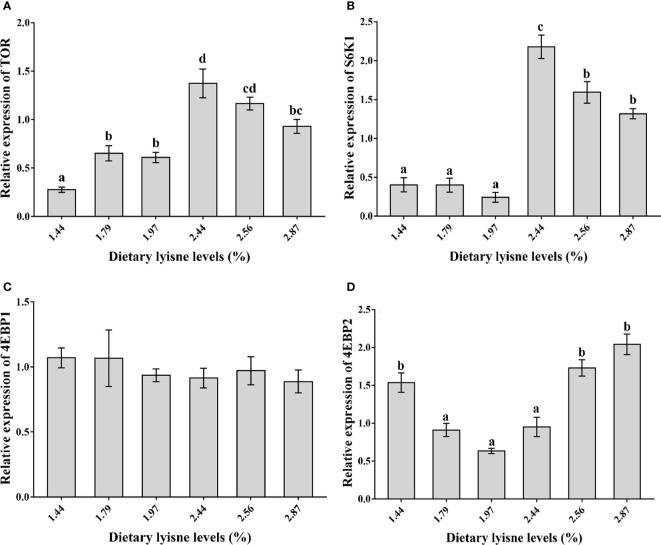
Relative mRNA expression of TOR signaling pathway in the intestine of grass carp fry. **(A)** Target of rapamycin (TOR); **(B)** S6 kinase1 (S6K1); **(C)** 4E-binding protein 1 (4EBP1); **(D)** 4E-binding protein 2 (4EBP2). Data are expressed as means with SEM. Values with different superscripts are significantly different (P < 0.05).

The relative mRNA expression levels of TOR, S6K1, 4EBP1 and 4EBP2 are assumed in **Figure 1**. From **Figures 1A, B**, it was shown that the TOR and S6K1 mRNA levels reached their maximum values when the fish were fed the 2.44% lysine diet and were markedly higher than those in the control group (*P* < 0.05). As the graded lysine levels increased from 1.44 to 1.97%, the 4EBP2 mRNA levels showed a decreasing trend and increased from 1.97 to 2.87%, and the minimum value was observed in the group fed the 1.97% lysine diet (*P* < 0.05; **Figure 1D**). Moreover, the 4EBP1 mRNA levels were not different among all the treatment groups (*P* > 0.05; **Figure 1C**).

### Relative mRNA Expression of Antioxidant Genes

**Figure 2:**
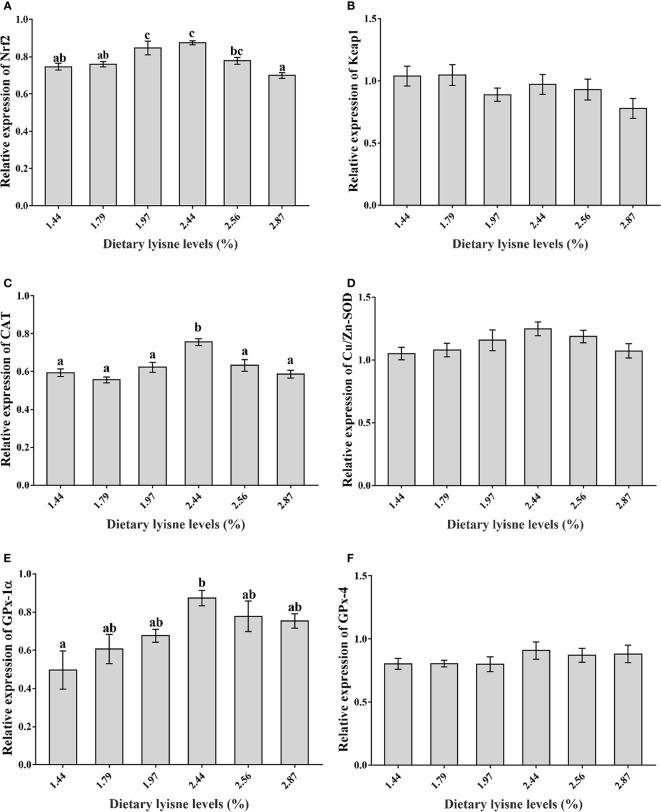
Relative mRNA expression of antioxidant genes in the intestine of grass carp fry. **(A)** Nuclear factor erythroid 2-related factor 2 (Nrf2); **(B)** Kelch-like ECH-associated protein 1 (Keap1); **(C)** Catalase (CAT); **(D)** Copper zinc superoxide dismutase (Cu/Zn-SOD); **(E)** Glutathione peroxidase 1*α* (GPx-1*α*); **(F)** Glutathione peroxidase 4 (GPx-4). Data are expressed as means with SEM. Values with different superscripts are significantly different (P < 0.05).

The relative mRNA expression levels of the antioxidant genes are assumed in **Figure 2**. **Figures 2A, C, E** show that the Nrf2, CAT and GPx-1*α* mRNA levels were markedly upgraded by increasing dietary lysine levels up to 2.44% (*P* < 0.05). Nevertheless, the Keap1, Cu/Zn-SOD and GPx-4 mRNA levels were not markedly affected by dietary lysine levels (*P* > 0.05; **Figures 2B, D, F**).

### Relative mRNA Expression of Immune Genes

**Figure 3:**
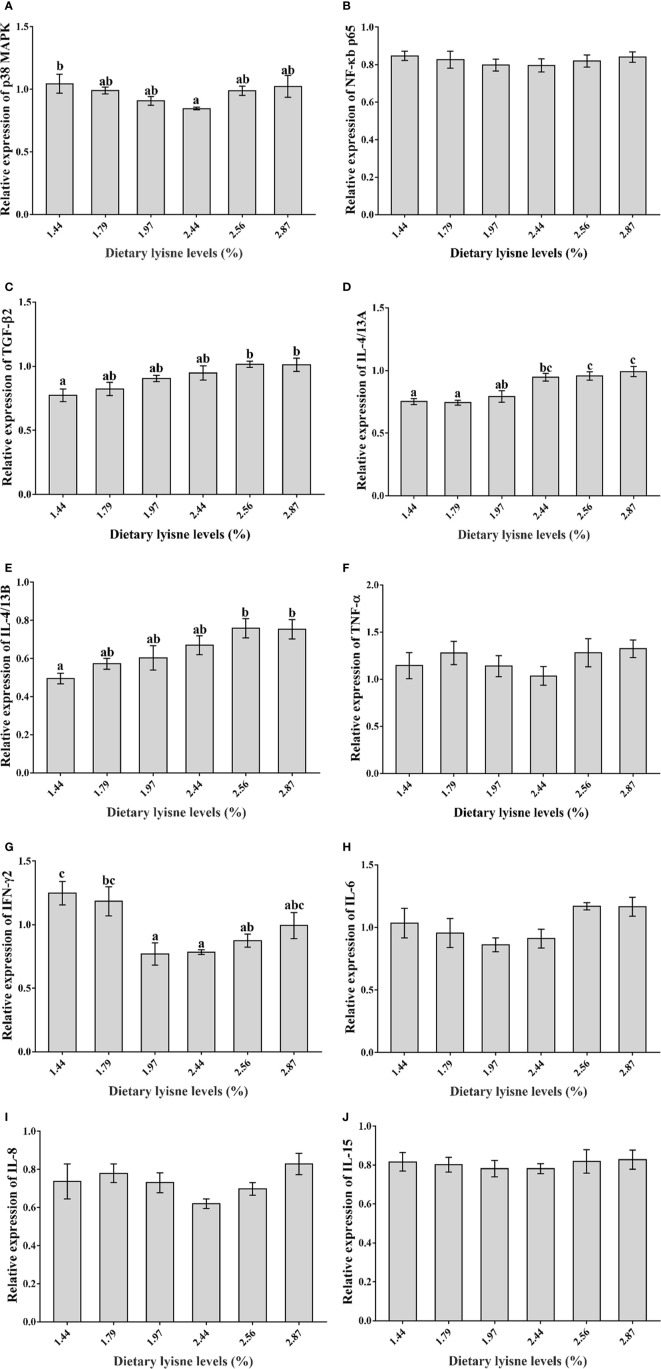
Relative mRNA expression of immune genes in the intestine of grass carp fry. **(A)** p38 mitogen-activated protein kinase (p38 MAPK); **(B)** Nuclear factor-kappa B p65 (NF-*κ*B p65); **(C)** Transforming growth factor *β*2 (TGF-*β*2); **(D)** Interleukin 4/13A (IL-4/13A); **(E)** Interleukin 4/13B (IL-4/13B); **(F)** Tumor necrosis factor α (TNF-α); **(G)** Interferon *γ*2 (IFN-*γ*2); **(H)** Interleukin 6 (IL-6); **(I)** Interleukin 8 (IL-8); **(J)** Interleukin 15 (IL-15). Data are expressed as means with SEM. Values with different superscripts are significantly different (P < 0.05).

The relative mRNA expression levels of the immune genes are shown in **Figure 3**. The minimum level of p38 MAPK was observed in the group of 2.44% lysine level (*P* < 0.05; **Figure 3A**). As anti-inflammatory cytokines, the a TGF-*β*2 and IL-4/13B mRNA levels in the intestines were significantly upregulated by 2.56 and 2.87% lysine levels compared with the control diet (*P* < 0.05; **Figures 3C, E**). Another anti-inflammatory cytokine, IL-4/13A, also showed the same trend; when the dietary lysine level reached 2.44%, the IL-4/13A mRNA expression levels were significantly increased (*P* < 0.05; **Figure 3D**). The minimum mRNA levels of the proinflammatory cytokine IFN-*γ*2 was observed in the fish fed 1.97% dietary lysine (*P* < 0.05; **Figure 3G**). Moreover, the NF-*κ*B p65, TNF-α, IL-6, IL-8, and IL-15 mRNA levels all presented the same trend as p38 MAPK and IFN-*γ*2; however, the difference was not significant among all lysine treatment groups (*P* > 0.05; **Figures 3B, F, H, I, J**).

### The Survival Rate of Grass Carp Fry

**Figure 4:**
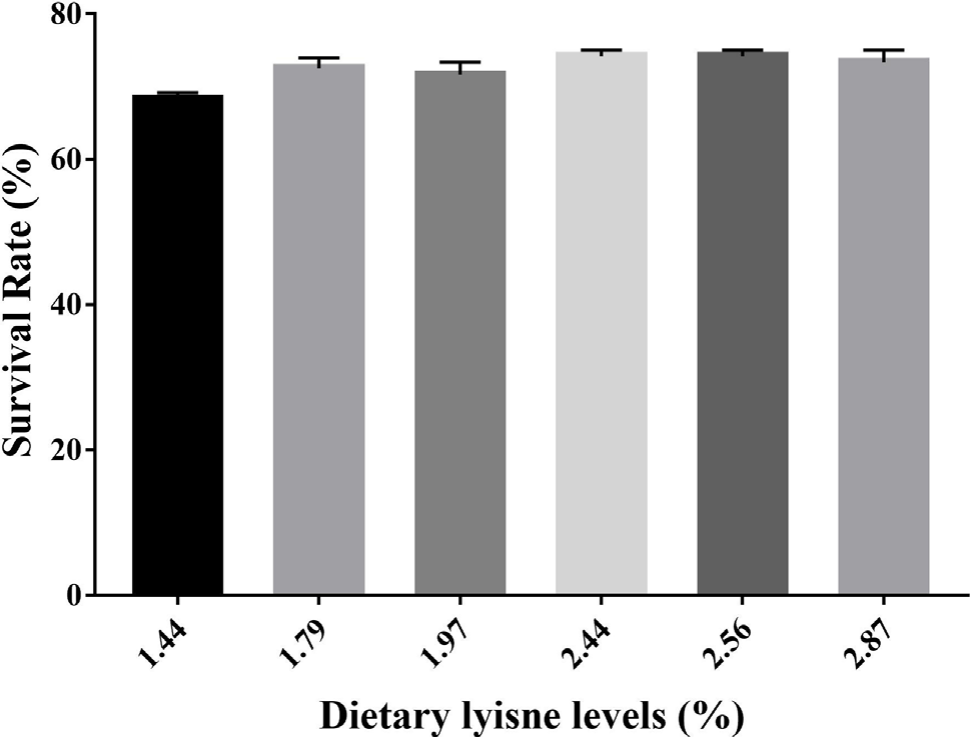
Survival rate (SR) of grass carp fry fed with different lysine levels. Survival rate (SR) (%) = 100 × (survival fish number/total fish number).

The survival rate (SR) of grass carp fry is assumed in **Figure 4**. The minimum SR was observed in the control group, and the fish fed with 2.44 or 2.56% dietary lysine levels exhibited the highest SR (*P* < 0.05).

## Discussion

Recent studies have shown that amino acids are involved in cellular immunity and humoral immunity in animals (41, 42). Lysine, one of the limiting amino acids, is also a signaling molecular that regulates the immune response (43). In mammals and fish, it was revealed that TOR could activate S6K1 and inhibit 4E-BPs, which then regulate anti-inflammatory cytokines (44, 45). In our experiment, the relative mRNA expression levels of TOR and S6K1 were markedly upregulated when fish were fed a 2.44% lysine diet compared with a control diet (1.44% dietary lysine). Moreover, the 4EBP1 and 4EBP2 mRNA levels were downregulated by 2.44% dietary lysine, which showed an opposite trend compared with TOR. Furthermore, the anti-inflammatory cytokines (TGF-*β*2, IL-4/13A and IL-4/13B) mRNA levels were markedly increased by high dietary lysine levels (2.44, 2.56, and 2.87%). These results suggested that appropriate lysine levels might activate anti-inflammatory cytokines to improve immune capacity *via* the TOR signaling pathway. As Song et al. (46) reported, TOR and S6K1 exhibit a positive correlation with the anti-inflammatory cytokines, which was consistent with our current results. In addition, it has also been indicated that TOR can inhibit 4EBPs to improve the immune response in the intestine of grass carp (31–34, 47), which was also presented in this experiment. However, the regulation mechanism of lysine on TOR signaling pathway needs to be further explored.

Several studies have reported that the p38 MAPK signaling pathway is closely associated with inflammation (48, 49), and related to the intestinal immune function of grass carp (36). Additionally, it was reported that activated p38 MAPK can facilitate the activation of NF-*κ*B pathway, which is an important activator of inflammatory processes and can regulate immune and inflammatory responses (50, 51). In our study, compared with the control group (1.44% dietary lysine level), the 2.44% dietary lysine level remarkably downregulated the p38 MAPK and NF-*κ*B p65 mRNA expression levels, which showed the same trend. These results revealed that appropriate lysine levels might promote the inactivation of p38 MAPK, which then might inhibit the expression of NF-*κ*B p65 and thus reduce the expression of proinflammatory factors (52). In addition, previous studies revealed that proinflammatory cytokines (IL-6, IL-8, and TNF-α, *etc*.) and anti-inflammatory cytokines (TGF-*β*2, IL-4/13A, and IL-4/13B, *etc.*) can both be regulated by the NF-*κ*B signaling pathway (53, 54). In rats, upregulation of the NF-*κ*B mRNA levels significantly increased the TNF-α mRNA levels in macrophages and the IL-8 mRNA levels in monocytes (11, 55). In humans, it has also been proven that the inhibition of NF-*κ*B p65 can inhibit the expression levels of proinflammatory cytokines (56), and the same results were observed in rabbits (57). The experimental results presented that the IFN-*γ*2 mRNA levels exhibited the same phenomenon as the NF-*κ*B p65 and p38 MAPK, and were significantly downregulated when fish were fed 2.44% dietary lysine. In addition, the mRNA levels of other cytokines downstream of the NF-*κ*B signaling pathway, namely, TNF-α, IL-6, IL-8 and IL-15, were also inhibited by 2.44% dietary lysine. As Li (18) reported, appropriate dietary lysine levels might reduce the expression of proinflammatory factors in the intestines by inhibiting the NF-*κ*B signaling pathway, which was consistent with our results. Furthermore, the NF-*κ*B signaling pathway is usually considered to be a proinflammatory pathway, so NF-*κ*B p65 is negatively correlated with anti-inflammatory cytokines (14), which could explain why the TGF-*β*2, IL-4/13A, and IL-4/13B mRNA levels were increased when NF-*κ*B p65 was downregulated in the fish fed with 2.44% dietary lysine. The results described above that dietary lysine could affect the intestinal expression of inflammatory cytokines *via* the p38 MAPK/NF-*κ*B signaling pathway in grass carp fry.

In fish, the antioxidant defense system mainly includes enzymatic and non-enzymatic systems (15). The enzymatic system is mainly composed of CAT, SOD and GPx, and the main representative of non-enzymatic systems is GSH (58). It was reported that amino acids can improve the antioxidant capacity of tissues and plasma in some fish species, a 0.40% dietary tryptophan level could increase the T-SOD, CAT activities, and the GSH content in blunt snout bream (*Megalobrama amblycephala*) (59). In this study, the group of 2.44% lysine level reached the maximum intestinal CAT and GPx activities, which were markedly higher than those of the control group. Moreover, the SOD activities and GSH contents showed increasing trends as the graded lysine levels increased from 1.44 to 2.44%. Our current results suggested that appropriate dietary lysine supplementation could improve antioxidant enzyme activities. As Li et al. (28) reported, 0.95 and 1.06% lysine levels could promote antioxidant defenses in the fish intestine by increasing the enzymatic antioxidant capacity and GSH content, which was in agreement with our findings. Furthermore, MDA reflects the degree of lipid oxidative damage (60). The intestinal MDA contents of grass carp fry in our study were not changed obviously; nevertheless, the MDA contents showed a downward trend when the graded lysine levels increased from 1.44 to 2.44%. Thus, according to the above results, 2.44% lysine supplementation could be beneficial for the intestinal health of grass carp fry.

The activities and gene expression levels of antioxidant enzymes are related to Nrf2-related signaling molecules (61). To date, the Nrf2-Keap1 signaling pathway is the most important endogenous antioxidative stress defense mechanism in cells, and Nrf2 and Keap1 play key roles in this signaling pathway (62). Nrf2 can affect downstream signaling pathways, which can also regulate the SOD, CAT, GPx and other antioxidant enzyme-related genes (63). In our experiment, the Nrf2 mRNA levels were markedly upregulated by 2.44% dietary lysine compared with control dietary lysine levels, and the Keap1 mRNA levels were not affected. These results showed that 2.44% dietary lysine may activate the Nrf2 signaling pathway. Similar to Nrf2, the mRNA levels of CAT and GPx-1*α* showed an increasing trend as the graded lysine levels increased up to 2.44%, and the maximum value was observed in the 2.44% lysine diet. The results described above indicated that the antioxidant enzymes gene expression levels and their related enzyme activities maintained the same trend, and both could be regulated by the Nrf2-Keap1 signaling pathway. In rats and fish, studies have shown that upregulating the Nrf2 mRNA levels and downregulating the Keap1 mRNA levels can promote the transcription levels of downstream antioxidant-related genes in liver (64, 65). In addition, it was speculated that lysine may regulate the intestinal Nrf2 mRNA expression to regulate the gene expression of downstream antioxidant enzymes and the activities of antioxidant enzymes in grass carp fry. These findings were in consistent with a previous study that reported that lysine could influence the Nrf2 signaling pathway in subadult grass carp (18).

On the other hand, as an upstream signaling molecule, TOR signaling positively modulates the Nrf2 signaling pathway (66). In our experiment, the TOR and Nrf2 mRNA levels presented similar trends, and the highest expression levels of both molecules were observed in the 2.44% lysine diet. This result was in agreement with a previous study which found that the upregulation of Nrf2 mRNA might be explained by the high expression of TOR (23). A study on cows reported the upregulation of p38 MAPK mRNA levels with decreasing Nrf2 mRNA levels in the mammary gland (67), which supported our findings in current study. Overall, 2.44% lysine supplementation could improve the intestinal antioxidant capacity of grass carp fry *via* the TOR, p38 MAPK and Nrf2 signaling pathways; however, the mechanism of by which dietary lysine regulates the TOR/Nrf2 and p38 MAPK/Nrf2 signaling pathways in grass carp fry is still unclear and requires further investigation.

In addition, most fish species have not yet fully developed their specific immune organs at the fry stage and cannot perform their own immune functions, thus, the survival rate of fry is often lower than that of adult fish (68). Thus, it is important to improve the survival rate in the fry stage. In this study, the survival rate of grass carp fry fed with 2.44% dietary lysine was higher than the control group (**Figure 4**), which suggested that 2.44% dietary lysine level could also increase the survival rate of grass carp in the fry stage by enhancing the immunity and antioxidant capacity.

**Figure 5:**
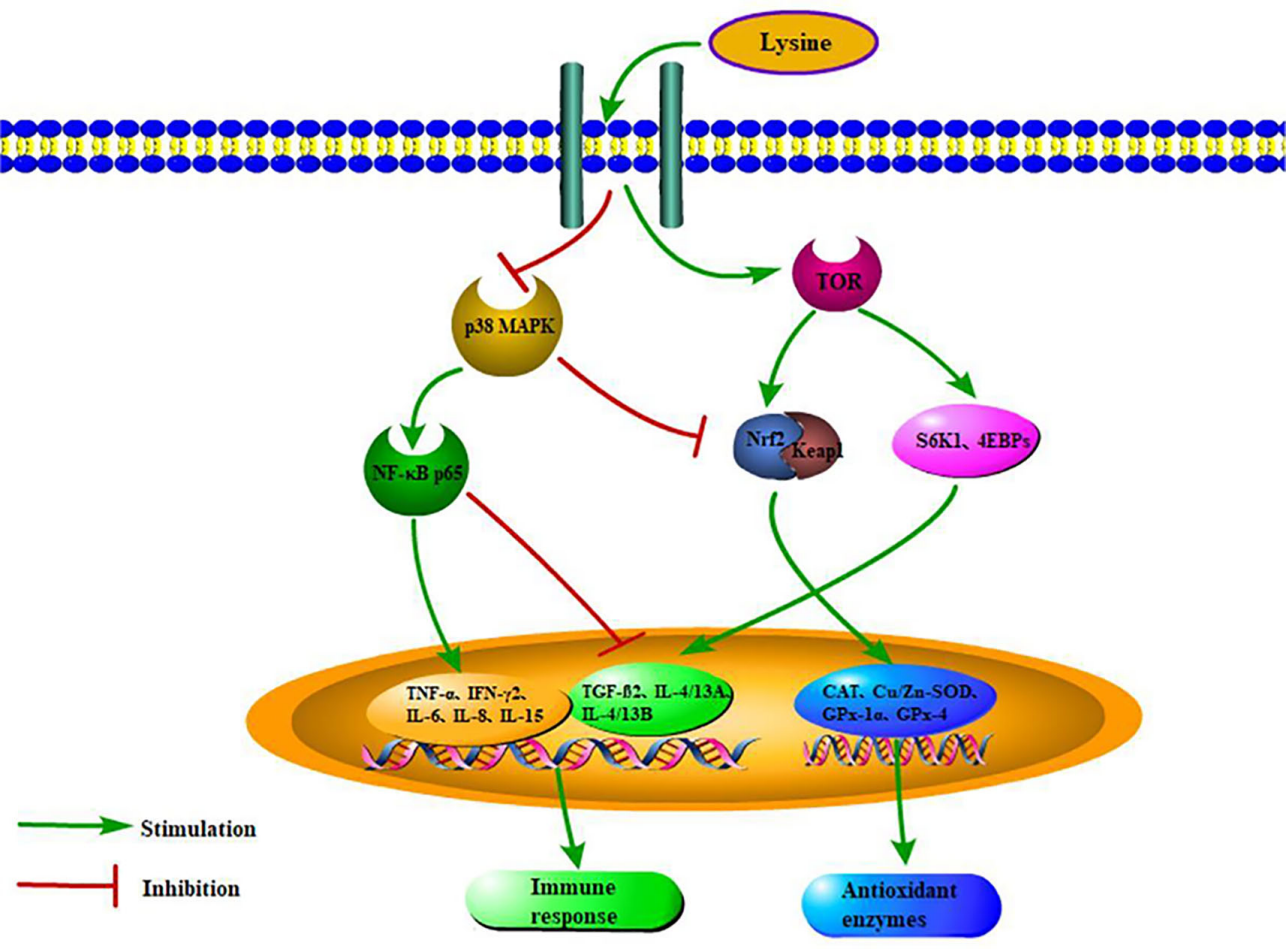
Scheme summarizing the mechanisms of optimal dietary lysine improved immunity and antioxidant status *via* TOR, p38 MAPK, Nrf2, and NF-*κ*B pathways.

In summary, our current study presented that dietary lysine levels may affect the intestinal health of grass carp fry by regulating the NF-*κ*B and Nrf2 signaling pathways through the TOR and p38 MAPK signaling pathways (**Figure 5**). The optimum dietary lysine level (2.44%) can promote intestinal antioxidant enzyme activities and upregulate the corresponding gene expression *via* the TOR/Nrf2 signaling pathway. Furthermore, 2.44% dietary lysine could also enhance intestinal immunity in grass carp fry by upregulating anti-inflammatory genes *via* the TOR/NF-*κ*B signaling pathway, and downregulating proinflammatory genes *via* the p38 MAPK/NF-*κ*B signaling pathway, which then improve the survival rate of grass carp fry.

## Data Availability Statement

The data analyzed in this study is subject to the following licenses/restrictions: The raw data supporting the conclusions of this article will be made available by the authors, without undue reservation. Requests to access these datasets should be directed to MR, renmc@ffrc.cn.

## Ethics Statement

The animal study was reviewed and approved and the handling of the fry was approved by the Institutional Animal Care and Ethics Committee of Nanjing Agricultural University, Nanjing, China (Permit number: SYXK (Su) 2011-0036).

## Author Contributions

MR and HL designed the study. DH carried out the experiments and wrote the manuscript. SM reviewed the manuscript. KJ and HY provided technical assistance. XG provided technical guidance. All authors contributed to the article and approved the submitted version.

## Funding

This study was financially supported by the National Key Research and Development Program of China (2018YFD0900400), the Natural Science Foundation of Jiangsu Province (BK20200169), National Natural Science Foundation of China, NSFC (31772820), and the Modern Agriculture Industrial Technology System special project of the National Technology System for Conventional Freshwater Fish Industries (CARS-45).

## Conflict of Interest

The authors declare that the research was conducted in the absence of any commercial or financial relationships that could be construed as a potential conflict of interest.
